# Extraordinary flight performance of the smallest beetles

**DOI:** 10.1073/pnas.2012404117

**Published:** 2020-09-21

**Authors:** Sergey E. Farisenkov, Nadejda A. Lapina, Pyotr N. Petrov, Alexey A. Polilov

**Affiliations:** ^a^Department of Entomology, Faculty of Biology, Lomonosov Moscow State University, Moscow 119234, Russia;; ^b^Southern Branch, Joint Russian-Vietnamese Tropical Research and Technological Center, Ho Chi Minh 70000, Vietnam

**Keywords:** flight, miniaturization, Ptiliidae, Coleoptera, insects

## Abstract

Size is a key to locomotion. In insects, miniaturization leads to fundamental changes in wing structure and kinematics, making the study of flight in the smallest species important for basic biology and physics, and, potentially, for applied disciplines. However, the flight efficiency of miniature insects has never been studied, and their speed and maneuverability have remained unknown. We report a comparative study of speeds and accelerations in the smallest free-living insects, featherwing beetles (Coleoptera: Ptiliidae), and in larger representatives of related groups of Staphylinoidea. Our results show that the average and maximum flight speeds of larger ptiliids are extraordinarily high and comparable to those of staphylinids that have bodies 3 times as long. This is one of the few known exceptions to the “Great Flight Diagram,” according to which the flight speed of smaller organisms is generally lower than that of larger ones. The horizontal acceleration values recorded in Ptiliidae are almost twice as high as even in Silphidae, which are more than an order of magnitude larger. High absolute and record-breaking relative flight characteristics suggest that the unique morphology and kinematics of the ptiliid wings are effective adaptations to flight at low Reynolds numbers. These results are important for understanding the evolution of body size and flight in insects and pose a challenge to designers of miniature biomorphic aircraft.

According to Tennekes’s “Great Flight Diagram,” flight efficiency considerably decreases with decreasing body size (1). Exceptions to this general rule are relatively few. At the same time, as the physical conditions of flight change during miniaturization, the forces of viscous friction become more prevalent, and the contribution of the drag forces increases. The structural and operational features of the wing apparatuses of microinsects are adaptations to flight at low Reynolds numbers (2). Most of the smallest insects have a feather-like (bristled) wing structure, known as ptiloptery (3, 4), in many cases independently evolved. Similar changes in wing kinematics and aerodynamics occur during miniaturization in many insect groups (5). The flight efficiency of the smallest insects in comparison with larger representatives of related taxa remained unknown.

The goal of our study was to analyze flight characteristics in ptilopterous microinsects and compare them with related larger species that have membranous wings. Our main model species were the smallest known free-living (nonparasitic) insects, featherwing beetles (Coleoptera: Ptiliidae). They belong to the infraorder Staphyliniformia (6) and were therefore compared with representatives of some of the phylogenetically closest families, Staphylinidae and Silphidae.

## Results

**Fig. 1.**
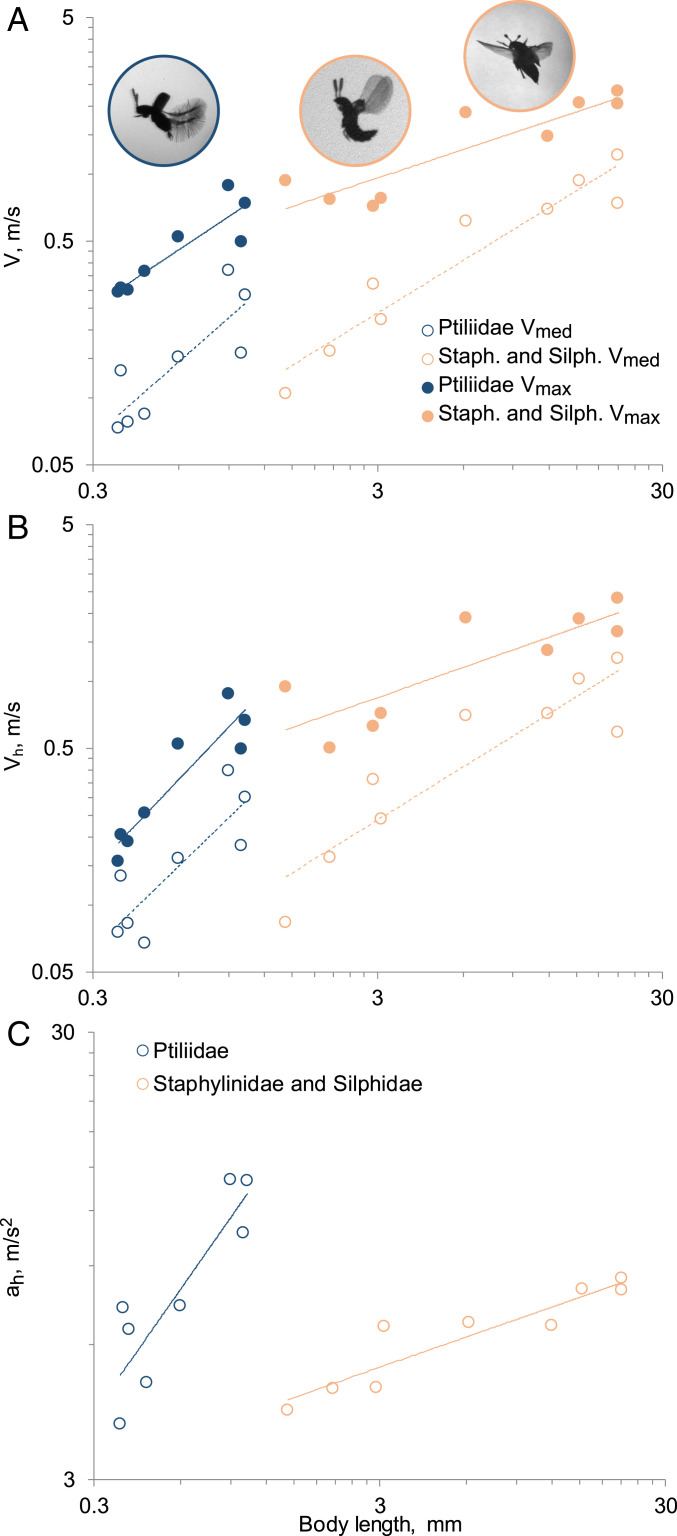
Flight parameters and body sizes of beetles: (*A*) median and maximum speed, beetles in flight (left to right): Ptiliidae, Staphylinidae, Silphidae; (*B*) median and maximum horizontal speed, color key as in *A*; (*C*) maximum horizontal acceleration.

The average (median) and maximum speed increases with increasing body length (BL) both in staphylinoids with membranous wings and in the ptilopterous Ptiliidae (Fig. 1 *A* and *B* and Table 1).

**Table 1.** Flight parameters of studied beetles

Family	Species	BL, mm	V_med_, m/s	V_med.h_, m/s	V_med.h.rel_, BL/s	V_max_, m/s	V_max.h_, m/s	V_max.h.rel_, BL/s	a_h_, m/s^2^
Ptiliidae	*Acrotrichis grandicollis* Mannerheim, 1844	1.03	0.29	0.30	293	0.73	0.66	645	13.97
	*A. sericans* Heer, 1841	0.90	0.37	0.39	437	0.88	0.88	975	14.05
	*Limulosella waspucensis* Hall, 1999	0.40	0.08	0.08	204	0.30	0.19	476	6.47
	*Mikado* sp.	0.46	0.08	0.07	146	0.36	0.26	556	4.92
	*Nanosella* sp.	0.37	0.07	0.07	202	0.30	0.16	421	3.97
	*Nephanes titan* Newman, 1834	0.60	0.15	0.16	267	0.52	0.52	870	7.33
	*Paratuposa placentis* Deane, 1931	0.38	0.13	0.13	351	0.31	0.21	540	7.25
	*Ptenidium pusillum* Gyllenhal, 1808	1.00	0.16	0.18	183	0.49	0.49	495	10.65
Staphylinidae	*Atheta* sp.	2.06	0.16	0.16	79	0.77	0.50	242	4.77
	*Dinaraea* sp.	3.11	0.22	0.24	78	0.77	0.71	229	6.58
	*Gyrophaena* sp. 1	1.43	0.10	0.08	58	0.93	0.94	658	4.28
	*Gyrophaena* sp. 2	2.92	0.32	0.36	123	0.71	0.63	214	4.80
	*Lordithon lunulatus* Linnaeus, 1760	6.16	0.61	0.70	114	1.87	1.91	311	6.72
	*Philonthus* sp.	11.97	0.69	0.71	60	1.46	1.36	114	7.93
Silphidae	*Nicrophorus investigator* Zetterstedt, 1824	21.07	1.21	1.26	60	2.34	2.33	111	6.61
	*Nicrophorus vespillo* Linnaeus, 1758	21.04	0.73	0.58	28	2.06	1.66	79	8.44
	*Oiceoptoma thoracicum* Linnaeus, 1758	15.39	0.93	1.01	66	2.07	1.89	123	8.00

Allometric analysis shows that the slopes of the median speed (V_med_) and median horizontal speed (V_med.h_) do not differ between the two groups (Fig. 1 *A* and *B*), while the elevation is significantly higher in Ptiliidae (−0.52 in Ptiliidae and −1.02 in other staphylinoids; Huber’s M estimation *P* < 0.001 for V_med_; −0.48 in Ptiliidae and −1.05 in other staphylinoids, *P* = 0.003 for V_med.h_), which shows that the speeds in ptiliids are higher than in other staphylinoids of comparable body sizes. The maximum speed (V_max_) and maximum horizontal speed (V_max.h_) correlate with the body size in both groups (Fig. 1 *A* and *B*). Pairwise comparison of the horizontal flight speeds of the ptiliid *Acrotrichis sericans* (which displays the highest speeds among Ptiliidae; V_max.h_ = 0.89 m/s) and four staphylinid species (*Dinaraea* sp., *Atheta* sp., *Gyrophaena* sp. 1, *Gyrophaena* sp. 2) shows that *A. sericans* has a higher median speed than staphylinids with bodies three times as long (Mann–Whitney *U* test, *P* < 0.001).

The maximum acceleration in Ptiliidae during horizontal flight (a_h_), reaching 14.05 m/s^2^, is significantly higher than in miniature Staphylinidae and almost twice as high as in the larger Silphidae, in which it does not exceed 8.44 m/s^2^ (Fig. 1*C*); the elevations of regression are significantly different.

The median speed of horizontal flight relative to BL (V_med.h.rel_) in Ptiliidae is 146 BL per s to 437 BL per s, higher than in the other staphylinoids (28 BL per s to 123 BL per s). The maximum horizontal flight speed (V_max.h.rel_) relative to BL is also mostly higher in Ptiliidae species (Table 1).

## Discussion

The average and maximum absolute speeds and maximum accelerations of Ptiliidae are comparable with those of much longer staphylinoids. Ptiliids are also comparable in flight characteristics with the much larger *Drosophila*, a model organism for studying insect flight (7). Relative values are even more extraordinary: *A. sericans* in horizontal flight cover up to 975 BL per s, which is the highest recorded relative horizontal speed of locomotion among animals.

Such outstanding values can be linked to two factors: structural features and kinematics of the wings, and size and efficiency of muscles. Physical characteristics of the musculature of Ptiliidae have not been studied, but it is known that miniaturization does not considerably increase relative volumes of muscles in beetles, in contrast to dipterans, where miniature species have membranous wings and very large flight muscles (8). The power of flight muscles depends on both volume and some structural features (9), but this aspect has not yet been studied in any ptilopterous insects, and the contribution of muscles to the flight efficiency of microinsects requires further study. The other productive way to increase flight efficiency is to optimize the structure and kinematics of the wings.

If the Reynolds numbers are low, the lift-to-drag ratio is very small both for membranous and for bristled wings of a similar shape (10) over the entire range of angles of attack; therefore, at low Reynolds numbers, it is more advantageous, in terms of energy, to use rowing flight, in which translational movements occur at a great angle of attack and create drag-based lift. Ptiliidae have bristled wings with peripheral setae covered with secondary outgrowths (11). This structure can reduce wing mass compared to that of a membranous wing of the same size, thus reducing inertial losses; at the same time, the permeability of bristled wings at low Reynolds numbers is very low, creating aerodynamic forces similar to those of membranous wings of the same size (10, 12). Bristled (compared to membranous) wings make use of the clap and fling mechanism more efficiently: smaller forces are required to draw such wings apart (13, 14); this is especially important, as the flight cycle of Ptiliidae includes not only the upper clap above the body but also the lower clap under the body (8). Features of this wing cycle may strongly increase aerodynamic efficiency: The wing moves back at a high angle of attack relative to the body and to the direction of movement during both upstroke and downstroke, while, in other miniature insects, the downstroke is directed forward and mostly creates drag-based lift (5).

Thus, the peculiar wing structure of Ptiliidae and peculiar features of their flight kinematics result in high flight efficiency despite minute size. Further study of the kinematics and aerodynamics of Ptiliidae can reveal new drag-based aerodynamic mechanisms. The ongoing miniaturization of artificial flying devices in electronics and robotics can make the knowledge of principles of flight aerodynamics of these and other microinsects useful for developing swimming and flying biomimetic devices.

## Materials and Methods

The method developed by us for studying flight characteristics of beetles is similar to earlier ones (15) but modified for studying miniature insects. We analyzed 17 species of staphylinoid beetles: eight Ptiliidae, six Staphylinidae, and three Silphidae (Table 1). Free flight was recorded using two Evercam 4000 synchronized high-speed cameras (Evercam) with a frequency of 60 frames per second (fps) to 300 fps. Between 5 and 30 individuals were placed in a closed transparent flight chamber illuminated from two sides and from above by 850-nm infrared LED. Additional visible light sources provided a natural level of illumination. The temperature in the chambers was 24 °C to 26 °C. The linear dimensions of the chambers exceeded the BLs of the beetles by a factor of at least 70, allowing the beetles to move freely and fly for up to several minutes. We have analyzed, on average, 9.76 tracks with an average duration of 561 frames (Dataset S1). We analyzed at least three records for each species, including a total of at least 492 track points. The flight trajectories were captured in two projections using Tracker (Open Source Physics). Three-dimensional reconstructions of trajectories, adjusted for scale and perspective, were made using visual markers on chamber walls or inside chambers. The distances between adjacent points of tracks did not exceed 15 BLs and were, on average, 1.36 BLs.

To remove tracking errors, the Cartesian coordinates of the tracks were smoothed after triangulation using local polynomial regression fitting, and calculated speeds were smoothed using moving average. The speeds and accelerations in horizontal sections of the trajectory (±30° relative to the horizon) were analyzed separately. The average value of each flight characteristic was calculated as the median for all values obtained for each species in each frame. Maximum speeds and accelerations were calculated as the 99th percentiles for all tracks for each species. Smoothing (stats package), descriptive statistics, and allometric analysis (smatr package) were performed in R. The scheme of the experimental setup and procedure of trajectory reconstruction are shown in Movie S1.

## Supplementary Material

Supplementary File

Supplementary File

## Data Availability

All study data are included in the article, Dataset S1, and Movie S1.
